# Supplementary material for: Oligomers of Carboxymethyl Cellulose for Postharvest Treatment of Fresh Produce: The Effect on Fresh-Cut Strawberry in Combination with Natural Active Agents
Source: Foods. 2022 Apr 13;11(8):1117. doi: 10.3390/foods11081117 (PMC9032414; doi:10.3390/foods11081117)
Supplement: Supplementary file 1 [file foods-11-01117-s001.zip › foods-1644378-supplementary.pdf]

Supplementary

Oligomers of carboxymethyl cellulose for postharvest treatment of fresh produce: The effect on fresh-cut strawberry in combination with natural active agents

Yevgenia Shebis [1,2], Elazar Fallik[3], Victor Rodov [3], Sai Sateesh Sagiri[1] and Elena Poverenov[1]*

[1] Agro-Nanotechnology and Advanced Materials Research Center, Department of Food Science, Agricultural Research Organization, The Volcani Institute, Rishon Lezion 7505101, Israel; yevgenia7777@gmail.com; elenap@volcani.agri.gov.il.

[2] The Robert H Smith Faculty of Agriculture, Food and Environment, The Hebrew University of Jerusalem, Rehovot 76100, Israel; yevgenia7777@gmail.com

[3] Department of Postharvest Science, Agricultural Research Organization, The Volcani Institute, Rishon Lezion 7505101, Israel; vrodov@volcani.agri.gov.il; efallik@volcani.agri.gov.il.

* Correspondence: elenap@volcani.agri.gov.il; Tel.: +972-3-9683354

Control O-CMC O-CMC vanillin

T2

T5

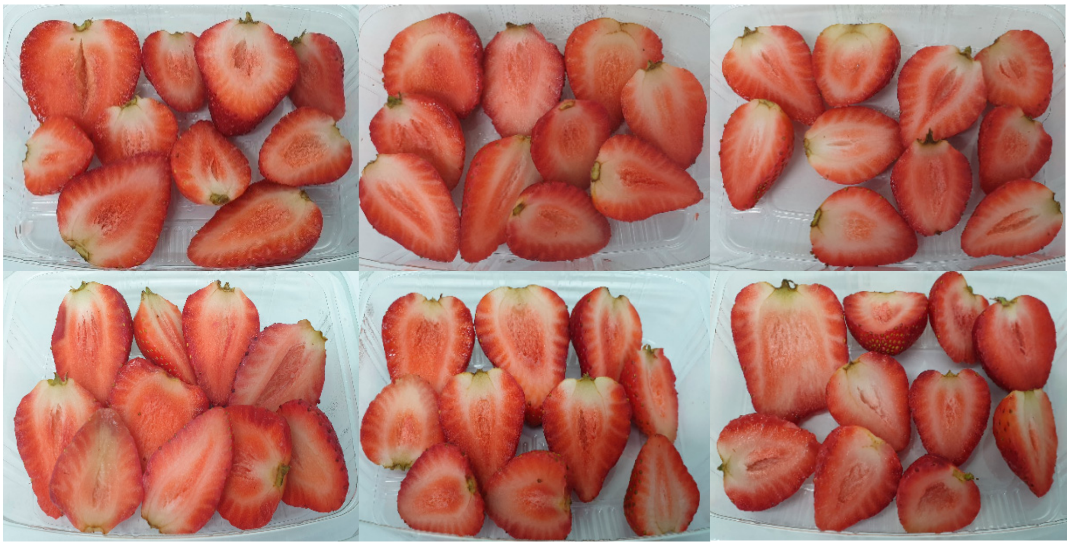

Figure S1: (Up, left to right) Untreated fresh cut strawberries (control); treated with O-CMC and O-CMC vanillin formulations after 2 days of storage at 4 °C. (Down, left to right) Untreated fresh cut strawberries (control); treated with O-CMC and O-CMC vanillin formulations after 5 days of storage at 4 °C.

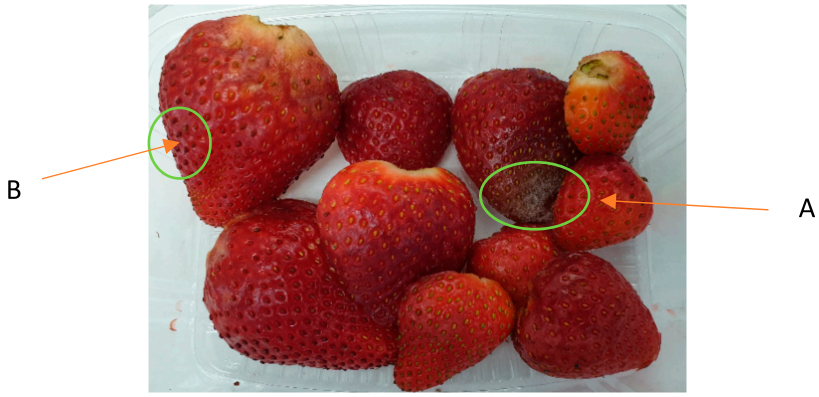

Figure S2: Decay incidents example of untreated fresh cut strawberries after 7 days of storage at 4 °C. A: small black spots.; B: big brown spots.
